# Microstructural and Tribological Characteristics of Composites Obtained by Detonation Spraying of Iron-Based Alloy—Carbide Powder Mixtures

**DOI:** 10.3390/ma16196422

**Published:** 2023-09-27

**Authors:** Fardad Azarmi, Xiangqing W. Tangpong

**Affiliations:** Department of Mechanical Engineering, North Dakota State University, Fargo, ND 58108, USA; annie.tangpong@ndsu.edu

**Keywords:** detonation gun praying, amorphization of coatings, ceramic-reinforced composite coatings, wear resistance

## Abstract

iron-based coatings have exhibited good mechanical properties, such as high hardness and good wear resistance, which are desirable properties in applications such as automobile brake rotors. iron-based coatings are also good replacements for Co- and Ni-based coatings, which are costly and could have health and environmental concerns due to their toxicity. In this research, three different iron-based coatings were deposited using the Detonation Gun Spraying (DGS) technology onto aluminum substrates, including the steel powders alone (unreinforced), and steel powders mixed with Fe_3_C and SiC particles, respectively. The microstructural characteristics of these coatings and mechanical properties, such as hardness and wear resistance, were examined. The morphology and structure of the feedstock powders were affected by the exposure to high temperature during the spraying process and rapid solidification of steel powders that resulted in the formation of an amorphous structure. While it was expected that steel particles reinforced with hard ceramic particles would result in increased hardness, instead, the unreinforced steel coating had the highest hardness, possibly due to a higher degree of amorphization in the coating than the other two. The microstructural observation confirmed the formation of dense coatings with good adhesion between layers. All samples were subjected to ball-on-disk wear tests at room temperature (23 °C) and at 200 °C. Similar wear resistances of the three samples were obtained at room temperature. At 200 °C, however, both ceramic reinforced composite samples exhibited higher wear rates in line with the reduction in their hardness values. This work explains, from the microstructural point of view, why adding hard particles to steel powers may not always lead to coatings with higher hardness and better wear resistance.

## 1. Introduction

Thermal spraying can be considered the most practical method of deposition of protective coatings on the surface of engineering components and parts. There are a number of commercialized thermal spraying methods, such as Atmospheric Plasma Spraying (APS), High Velocity Oxygen Fuel (HVOF), Wire Arc Spraying (WAS), Vacuum Plasma Spraying (VPS) and Detonation Gun Spraying (DGS) [1,2,3,4,5,6]. All these techniques are widely used to deposit different materials, such as ceramics, metals, alloys, and cermet, on the surfaces of engineering parts [5,6,7,8] that operate in harsh environments, i.e., highly erosive, corrosive, or extremely high temperature service conditions, for example, dies, drill bits and nozzles in high-operating temperatures, oil pipes, etc. [3,4,6,9]. Among the various thermal spraying methods, DGS is considered one of the most available and economical deposition techniques due to its simplicity and ability to produce coatings with relatively good mechanical properties [6,7,8,10,11,12,13]. The fundamental features of this technology are: (1) the explosion of a mixture of gases such as oxygen and acetylene inside the gun barrel triggered by a spark, (2) the feedstock powders are introduced into a hot chamber, melted and accelerated toward the substrate, and (3) the molten particles impact on the substrate and build up the coating. Generally, DGS-deposited coatings exhibit low porosity levels close to that of the bulk materials, high bonding strength between splats and high adhesion between the coating and substrate [14]. These advantages are the result of the relatively high in-flight speed of the accelerated powders (up to 800 m/s) and relatively low processing temperature [15,16]. Therefore, the spraying time is less and the sprayed powders experience a lower degree of decomposition, i.e., formation of undesired phases during the deposition process, unlike the APS or HVOF processes that could have a higher degree of decomposition [17,18,19].

The possibility of deposition of iron-based coatings via thermal spraying has always been attractive for scientists and industry [20,21]. The iron-based coatings have exhibited good combination of the mechanical properties such as hardness and wear resistance [22,23,24,25]. Another reason for the growing interest in iron-based coatings is to replace Co- and Ni-based coatings, which have complications in application due to their toxicity, high price, requirement of the addition of a binder phase for stability, and the possibility of allergic skin reactions in the case of Ni-based alloys [20,21,22,23,24]. It is speculated that the performance of iron-based coatings in erosive and harsh environment service conditions, such as marine pumps, could also be improved with the addition of some hard materials to the steel powder matrix, such as carbides, nitrides, and borides [1,2,26,27]. Fe_3_C and SiC have been considered as the reinforcement phases in this study due to their high hardness, good wear resistance, and low cost, especially compared to borides and nitrides [28,29]. However, limited research has been conducted on the iron-based composite coatings with hard particle reinforcements [22,29], and, particularly, there is a lack of information about characteristics of such steel coatings produced using thermal-spraying technology. This study examines the properties of such coatings produced using commercially available steel powders and steel powders reinforced with Fe_3_C and SiC particles by the DGS method. The goal is to investigate the microstructure and mechanical properties of deposited coatings to address their relationship. DGS as a cost effective and relatively less complicated thermal spray technology can be considered a good candidate for the production of steel composite coatings upon successful results.

Steel composites can be deposited on a variety of metallic surfaces to improve their mechanical strength. Aluminum and aluminum alloys possess high specific strength and low weight [30,31]; meanwhile, they have exhibited poor sliding properties and low wear resistance [30,32,33]. Thus, it is worthwhile to find a solution for these shortcomings of aluminum-based engineering parts and components. Therefore, aluminum has been selected as the substrate material for all coatings in this study.

## 2. Materials and Methods

Three types of powders (100 wt.% SHS-7574, SHS-7574+50 wt.% Fe_3_C and SHS-7574+50 wt.% SiC) were used to deposit 3 types of coating samples in this study. The SHS-7574 powder was produced via gas atomization by Nanosteel, RI, USA. The nominal composition of SHS-7574 powder was Cr < 25%, Mo < 20%, W < 10%, B < 5%, Mn < 5%, C < 3%, Si < 2% and Fe ball (wt.%). The commercially available Fe_3_C and SiC powders suitable for thermal spraying (Plackart, Moscow, Russia) were mixed with 50 wt.% SHS-7574 powder was used to make two different composite samples. The size distribution and nominal composition of Fe_3_C and SiC powders were determined experimentally, since this information was not provided by the powder manufacturer. While some unmelted or partially melted hard ceramic particles may have shattered during impact, there was no evidence of their decomposition after the spraying process. Plates of aluminum alloy-2024 with hardness of 111 ± 9 HV and dimensions of 20 mm× 20 mm× 2 mm were used as substrates for the deposition of all coating coupons in this study. The surfaces of the substrates were cleaned and roughened by sandblasting using Al_2_O_3_ particles (120 grit) prior to spraying. The powders were sprayed onto a substrate by using a DGS equipment (Ob DGS, Plackart, Russia). Each layer of the coating was deposited with an approximate thickness of 6 µm during the spraying process. All coating samples were deposited with thicknesses in the range of 800–1000 µm. The spraying parameters of DGS are listed in Table 1. In this study, three different names are used to distinguish the coating samples: “Steel” for 100 wt.% SHS-7574 powder and coating, “St-Fe_3_C” for SHS-7574 + 50 wt.% Fe_3_C and “St-SiC” for SHS-7574 + 50 wt.% SiC.

The phase compositions of powders and deposited coatings were analyzed by X-ray diffractometry (XRD) using X’PERT PRO (PANalytical, The Netherlands) with Cu-Kα irradiation. A scanning was performed for each sample at 2θ ranging from 5° to 100° with a scanning step of 0.05° at operating voltage of 40 kV and current of 40 mA. The XRD patterns were analyzed using STOE WinXpow version 1.04 to identify the actual phase composition of the powders and deposited coatings.

The cross-sections of the deposited coating were cut and mounted into the PolyFast Bakelite. Due to significant differences in hardness of matrix and reinforcing phase, special attention and detailed polishing were required to obtain a smooth surface appropriate for metallography on reinforced samples. The grinding process was completed using different grades of sandpapers with 9 μm, 3 μm, 1 μm, and 0.04 μm suspensions. The morphology of the initial powders and coating cross-sections were investigated by scanning electron microscopy (SEM) using a Phenom XL (The Netherlands) equipped with energy dispersive X-ray analysis (EDS) module. The particle size distribution of feedstock powders was determined using the Digimizer 4 software for image analysis. Statistics included complete measurements for a minimum of 500 particles for each type of powder. Image analysis for porosity measurement on the cross-section of coating samples was conducted on seven SEM micrographs randomly taken from different regions of the microstructure at the same magnification.

The hardness of coatings was measured on the polished cross-section using Emcotest DuraScan-20 G5 (Struers, Cleveland, OH, USA) with a 200 g load and exposure time of 35 s. The bulk hardness of coatings and hardness at the coating substrate interface were measured at 10 random points and then averaged after excluding the maximum and minimum recorded values.

The wear behavior of coatings was tested using a ball-on-disk Tribometer (CSM Instruments/Anton Paar, Aarau, Switzerland) against a sintered α-Al_2_O_3_ ball of a diameter of 6 mm at 23 °C and 200 °C in dry conditions following ASTM G99-17 [34]. The samples had dimensions of 20 × 20 mm. The applied normal load was 20 N, the linear relative sliding speed was 0.26 m/s and the overall sliding distance was 1000 m. The wear test was performed in a unidirectional straight line due to the size limitation. The standard grinding and polishing procedures were used to prepare the top surface of the coating samples before the wear tests. The roughness of surface before and after wear tests were measured using a Hommel Tester T8000-RC120-400 (JENOPTIK Industrial Metrology, Villingen-Schwenningen, Germany). The wear rate was calculated from examining the profile of the wear track measured by a profilometer. The wear tracks on the top surface of coatings were also investigated by SEM using a JEOL JCM-6000 microscope (JEOL, Tokyo, Japan).

## 3. Results

### 3.1. XRD of Feedstock Powders and DGS Deposited Coatings

**Figure 1:**
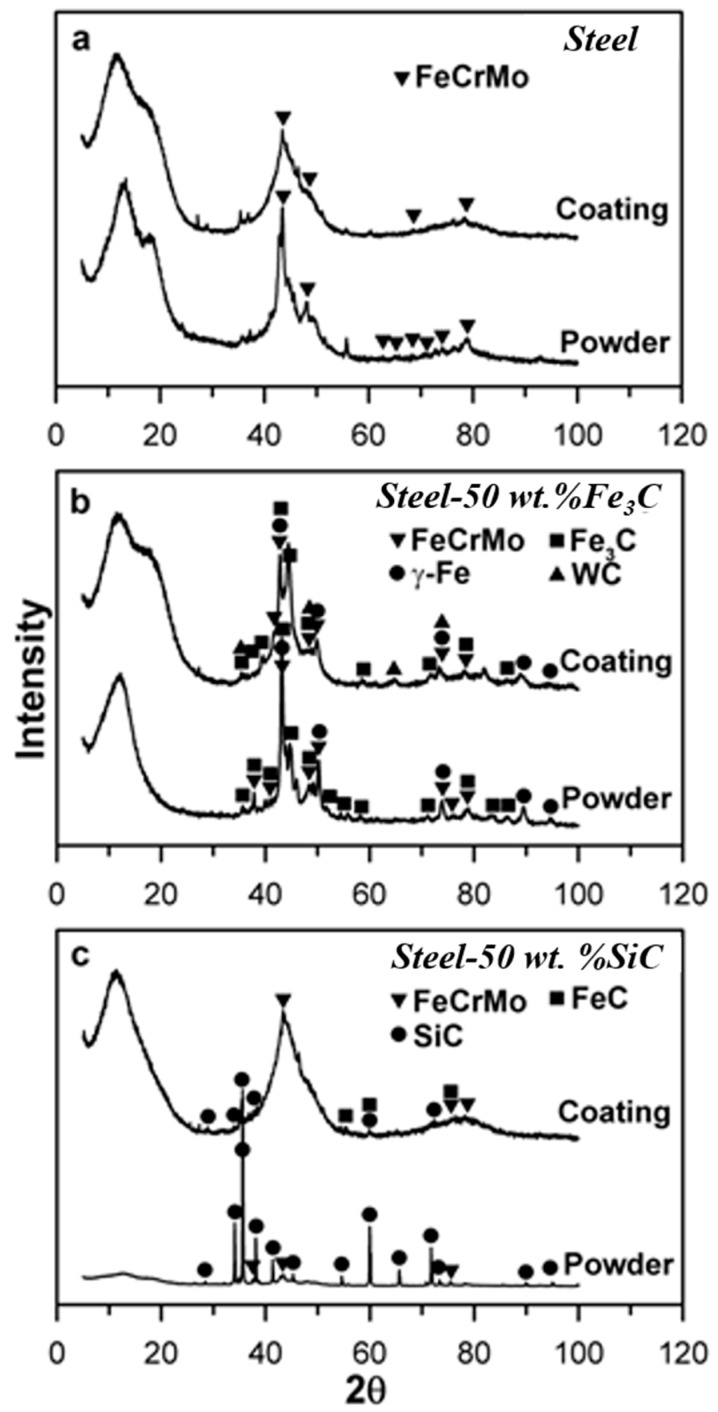
The XRD patterns obtained from feedstock powders and coatings of (**a**) Steel, (**b**) St-Fe_3_C, and (**c**) St-SiC.

Figure 1a–c show the results of XRD analysis for steel, St-Fe_3_C and St-SiC feedstock powders and the corresponding DGS deposited coatings. Figure 1a shows clearly that the steel powder mainly contains one major phase of FeCrMo. The wide peaks in the XRD pattern of steel powder indicate the existence of an amorphous structure. Usually, the powder produced using gas atomization possesses some degree of amorphization [13,35,36]. The steel coating deposited via DGS was characterized by an almost fully amorphous structure with some small peaks belonging to the FeCrMo crystalline phase as shown in Figure 1a. 

The St-Fe_3_C initial powder exhibited more complex phase composition compared to pure steel powder as presented in Figure 1b. The St-Fe_3_C powder consists of the γ-Fe, and Fe_3_C phases, in addition to the FeCrMo phase. The phase composition of St-Fe_3_C coating consists the same γ-Fe, Fe_3_C, and FeCrMo peaks, confirming that the DGS deposition process had no significant effect on the phase composition of the initial powder. In addition to the major phases, some negligible traces of WC were observed in the XRD pattern of St-Fe_3_C coating. It shows that formation of some amorphous phases may have occurred during DGS deposition as well. 

It is clear from Figure 1c that the initial powder of St-SiC consists of two major phases, SiC and FeCrMo. It is worth mentioning that all peaks were sharp, which was an indication of crystallinity in the initial powder. In addition, it seems that the intensity of the SiC and other detected phases could overpower the major peaks that belong to steel as seen in Figure 1a,b. The XRD analysis of the coating revealed that a large amount of amorphous phase was formed during the DGS deposition process. Previous studies have also confirmed formation of such amorphous phases possibly due to rapid cooling during thermal spraying processes where they could not detect any distinguished peaks from crystalline phases in deposited coatings of similar materials [36,37]. Despite SiC and FeCrMo phases, some peaks from FeC were also detected in the XRD pattern of St-SiC coating.

### 3.2. Microstructure (Feedstock Powder and Coatings)

**Figure 2:**
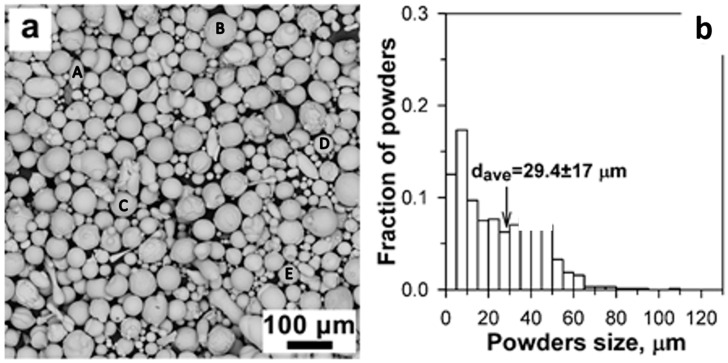
(**a**) Morphology of the original steel powder, and (**b**) particles size distribution obtained by SEM. The inset letters indicate the location of the EDS analysis as listed in Table 2.

The morphology and particle size distribution of the steel powder are shown in Figure 2a. The dominant powder geometry was spherical; however, some elongated particles were also observed in the microstructure that are indications of gas atomized particles [36]. An average particle size was measured to be 29.4 ± 17 μm as shown in Figure 2b.

The actual chemical composition of the powders was measured using EDS analysis. Since the initial feedstock powders in this study were produced by mixing different materials, to better distinguish particles and composition distribution, the results of point detection are listed in Table 2. It is worth mentioning that the EDS was not capable of identifying oxygen in the coating composition due to the detection limit of the EDS equipment.

**Figure 3:**
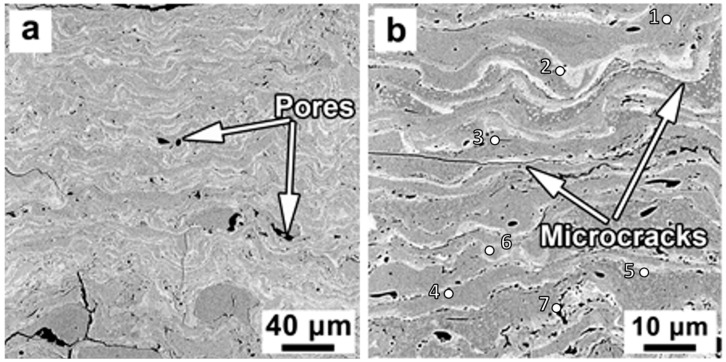
SEM micrographs of the unetched cross-section of the DGS deposited steel coating at (**a**) low magnification, and (**b**) high magnification. The inset numbers indicate the location of the EDS analysis. The EDS results are summarized in Table 2.

The SEM images of an unetched cross-section of the steel coating are presented in Figure 3. The lamellae-shaped microstructure, which is typical for thermally sprayed coatings, is present. The change of contrast is clear in Figure 3 at different layers of the microstructure, which is an indication of differences in phases or chemical compositions. The brightest areas (Table 2, points two, and four to seven) were enriched by tungsten, the amount of which was varied from 5.02 to 12.58 wt.%. While the gray areas (Table 2, points one and three) were characterized by a higher amount of Fe compared to the other areas. The darkest regions in the microstructure are identified as pores. The measured porosity of steel coating was 4.95 ± 0.70 Vol.%, indicating the deposited coating was dense and of high quality.

The morphology of the pores varied from round shape to irregular ones (Figure 3b). It has been found that the sphere-shaped pores were mostly due to the gas entrapment during impact, flattening and solidification of splats [4], and the elongated pores were formed mostly between splats along with microcracks. Generally, the development of microcracks has been closely related to the high internal stress in the coating due to high in-flight particle velocity or high temperature gradient during the solidification process [3,4]. 

**Figure 4:**
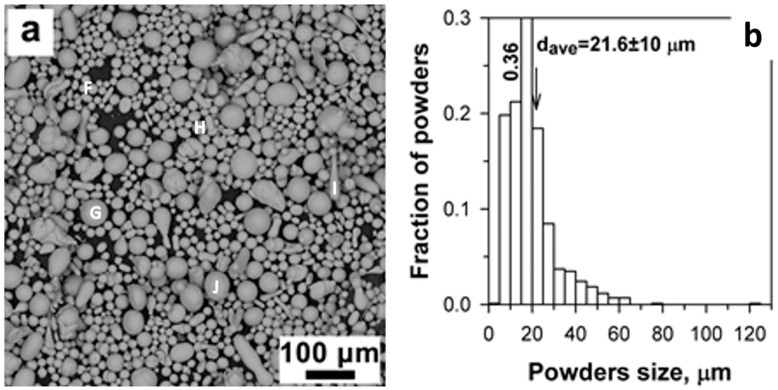
(**a**) Morphology of the original St-Fe_3_C powder, and (**b**) particles size distribution by SEM. The inset letters indicate the location of the EDS analysis. The EDS results were summarized in Table 3.

Figure 4 presents the morphology and particle size distribution of St-Fe_3_C powders. Most of the powder particles are in spherical shape, and there is a small amount of elongated ones. The particle size distribution was characterized by a relatively wide profile as shown in Figure 4b. The average powder size of 21.6 ± 10 μm was slightly smaller than the steel powder size (29.4 ± 17 μm). The chemical composition analysis performed on the points F, G and H indicated similar composition as the steel powders as shown in Figure 4a and Table 3. However, traces of Fe carbides were also detected at points H and I as listed in Table 3.

**Figure 5:**
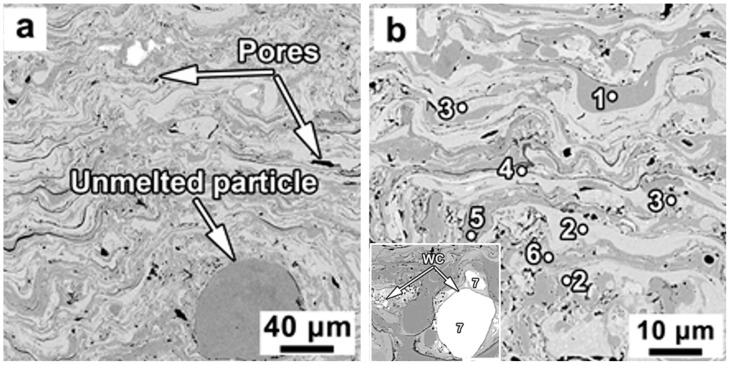
SEM micrographs of the unetched cross-section of the DGS deposited St-Fe_3_C coating at (**a**) low magnification, and (**b**) high magnification. The numbers indicate where the EDS analysis was performed. The results of EDS analysis were summarized in Table 3.

Figure 5 shows the SEM images of the unetched cross-section of the St-Fe_3_C coating. The DGS resulted in formation of well-defined layered structure identified by different contrasts (Figure 5a,b. The difference in chemical composition of the phases was identified using EDS analysis. The point one region was found to be enriched by Fe (~97 wt.%) with a small amount of Cr and C (Table 3), i.e., this phase could represent iron carbide. The light gray regions (points two and three) basically represent the steel powders according to the measured chemical composition (Table 3). Region marked as three was enriched by W identified by lighter contrast compared to region two as shown in Figure 5b. The middle gray regions were characterized by increased iron content of more than 70 Wt.%. The formation of iron-enriched regions may be due to the interaction of molten steel and iron carbide powders during the deposition process. In addition to some unmelted iron carbide as marked in Figure 5a, the pure WC was detected in the microstructure as well (Table 3, point seven). The size of these WC particles varied from 1 to 20 μm (inset in Figure 5b). Again, black regions were identified as pores in (Figure 5a,b). These pores were characterized by irregular shapes with an average size of 5 ± 1.0 μm and there were some with elongated shapes along the splat boundaries. The porosity of the St-Fe_3_C coating was 2.24 ± 033 vol.%, which is approximately half of that of the steel coating. It appears that small Fe_3_C particles could fill the voids and pores in the microstructure. Iron is the common element, which could result in a higher diffusion rate and, consequently, formation of a strong bond between matrix and reinforcement phases.

**Figure 6:**
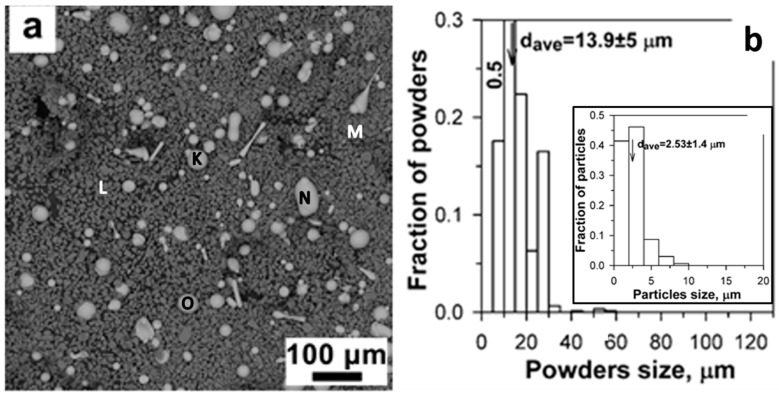
(**a**) Morphology of the original SHS-7574 + SiC powder, and (**b**) particle size distribution by SEM. The inset letters indicate the location of the EDS analysis. The EDS results were summarized in Table 4.

The SEM micrograph of St-SiC powder and particle size distribution are shown in Figure 6a,b. The initial powder contained two clearly distinguished types of particles, spherical shapes in light gray color, and irregular shapes in dark gray. The light gray particles were mixtures of several elements (points K, N and O in Table 4), while the small dark gray particles were found to be pure silicon carbide (points M and L in Table 4). The particle size distribution was concentrated in the range from 2 to 30 μm and the average size of the particles was 13.9 ± 5 μm (Figure 6b), indicating the influence of smaller SiC particles. The inset of Figure 6b is the particle size distribution of SiC particles in St-SiC feedstock powder.

**Figure 7:**
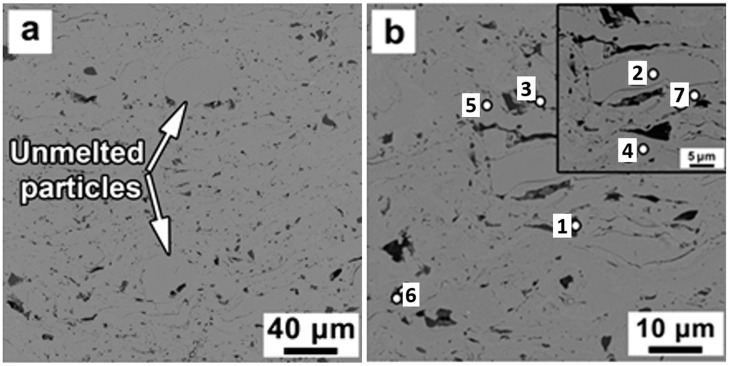
SEM micrographs of the unetched cross-section of the St-SiC coating produced via DGS at (**a**) low magnification, and (**b**) high magnification. The inset numbers indicate the location of the EDS analysis. The EDS results were summarized in Table 4.

The microstructure of St-SiC coating contains unmelted particles surrounded by matrix as labeled in Figure 7a. The unmelted particles appear to be in spherical shapes mainly consisting of steel particles. Note here that the matrix was characterized by uniform atomic number contrast in comparison with a matrix of steel and St-Fe_3_C coatings, where more variations of gray scale were observed (Figure 3 and Figure 5).

The EDS analysis at points two, four, five, and seven revealed negligible differences in actual chemical composition (Table 4). The dark regions (points one, three, and six) were identified as SiC in irregular shapes with an average size of 2.95 ± 1.72 μm, close to the average size of 2.53 ± 1.4 μm for SiC in the inset of Figure 6b. The porosity of the St-SiC coating was 1.07 ± 0.44% vol.%, which is approximately half of that of the St-Fe_3_C coating.

### 3.3. Mechanical Properties

#### 3.3.1. Hardness

**Figure 8:**
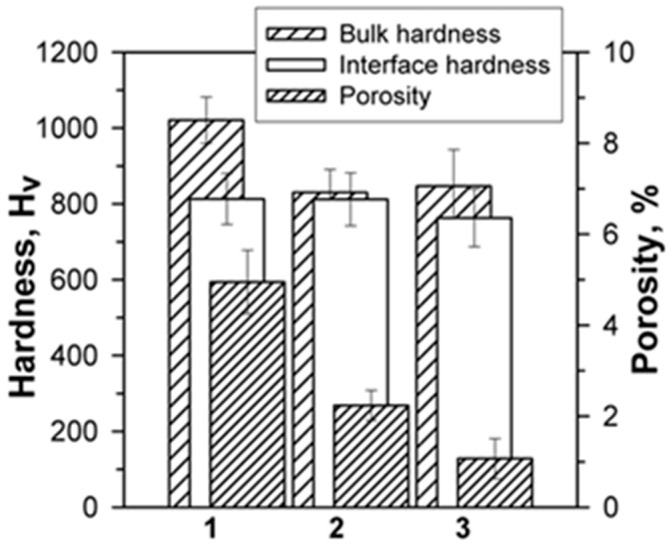
The hardness (bulk and interface) and porosity of (1) steel, (2) St-Fe_3_C, and (3) St-SiC coatings deposited by DGS in this study.

The measured hardness values of coatings (bulk hardness) and coating/substrate interfacial regions, and porosity distributions in the coatings are presented in Figure 8. The steel coating has the highest bulk hardness instead of having the highest porosity, although its porosity was still considered fairly low. The measured hardness for St-Fe_3_C and St-SiC coatings were lower than the steel coating. Table 5 shows the prediction hardness according to the rule of mixture. As stated before, the original assumption based on hardening effect was that the coating hardness would increase with the additions of hard phases; however, the hardness results indicate the opposite. These results will be discussed in detail in the next sections.

The porosities of both reinforced composite coatings are significantly lower than that of the steel coating. However, the three sets of hardness values for the coating/substrate interfacial areas are remarkably close. The similar coating/substrate interfacial hardness values indicate similar interfacial strength of these coatings, possibly due to the low porosities in the coatings. 

#### 3.3.2. Wear Test

The friction coefficients and wear rates are shown in Table 6. It is clear that the St-SiC coating has the largest friction coefficient at room temperature. The lowest friction coefficient of 0.479 was recorded for St-Fe_3_C coatings and 0.564 for the unreinforced steel coating. The wear rate of the St-SiC coating, 0.17 mm^3^/h, is also the highest among all samples. Note that the wear rate of the steel coating (0.13 mm^3^/h) was lower than that of the St-Fe_3_C coating (0.15 mm^3^/h) although it had higher coefficient of friction. 

An increase in the test temperature to 200 °C resulted in an interesting behavior of the friction coefficient for steel and St-SiC coatings. The former experienced a significant decrease in the friction coefficient from 0.564 to 0.04. In addition, the wear rate of the steel coating was also considerably decreased from 0.13 mm^3^/h to 0.03 mm^3^/h. The St-SiC coating had a slight reduction of the friction coefficient from 0.745 to 0.657. However, the St-Fe_3_C coating had an increase of the friction coefficient from 0.479 to 0.584. The wear rates of both composite coatings were significantly increased by approximately three times when the test temperature was increased to 200 °C. Note that the St-SiC coating had the highest friction coefficient at both temperatures. 

**Figure 9:**
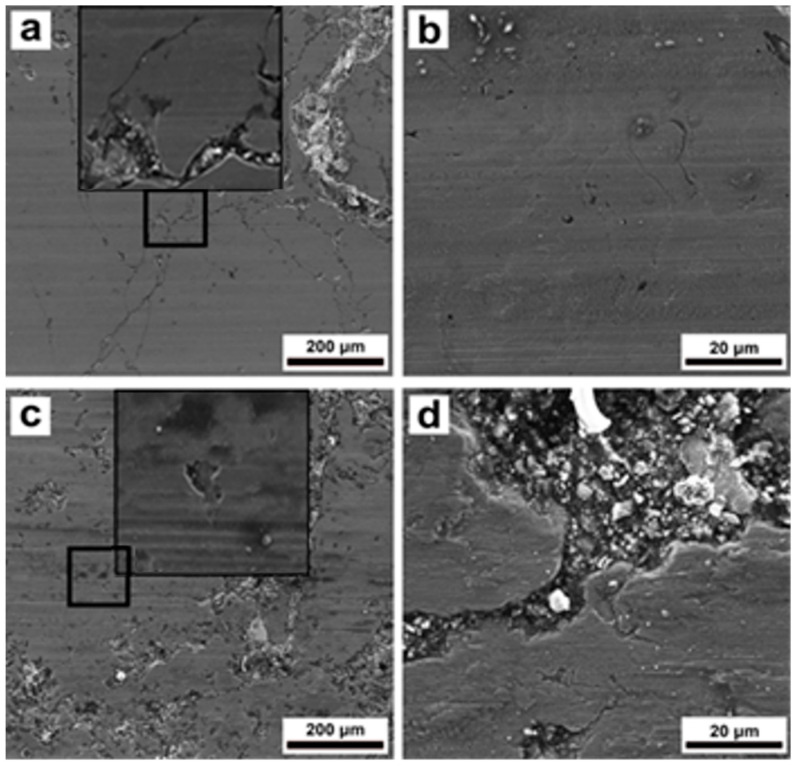
SEM micrographs of the wear tracks on steel sample after wear tests of DGS deposited steel coating at 23 °C (**a**,**b**), and 200 °C (**c**,**d**).

Figure 9a–d show the wear tracks formed on the surface of the steel coating during the wear tests at 23 °C and 200 °C. At the first glance, the wear tracks were characterized by a relatively smooth surface after the test at the ambient temperature (Figure 9a,b). However, higher magnification observation in Figure 9a indicated the existence of microcracks on the steel coating surface. Microcracks could occur due to the high internal stresses as a result of rapid solidification of the molten particles during the spraying process [4].

Another reason for the development of microcracks could be crushing of the surface by hard balls during the wear test. The steel coating tested at 200 °C was severely damaged compared to the sample tested at room temperature (Figure 9a,c). The relatively large microcracks and ruts were observed on the worn surface of the sample tested at 200 °C (Figure 9c). Some debris was also observed on the worn area as shown in Figure 9d. 

**Figure 10:**
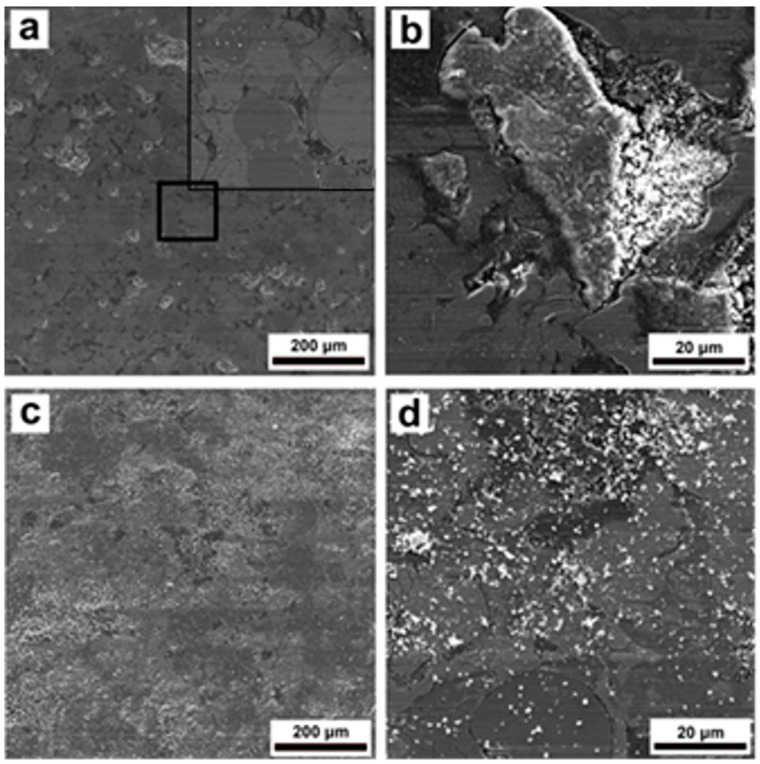
SEM micrographs of the wear tracks after wear tests of DGS deposited St-Fe_3_C coating at 23 °C (**a**,**b**), and 200 °C (**c**,**d**).

The microstructures of worn surfaces of the St-Fe_3_C coating are presented in Figure 10a–d. Compared to the steel coating, the St-Fe_3_C coating had a higher degree of damage with many grooves on the worn surface (Figure 10a). Most of those channel type features were formed on round-shaped unmelted Fe_3_C particles as shown in the inset of Figure 10a. Generally, these unmelted ceramic particles exhibit lower adhesion with the bulk coating. Furthermore, the St-Fe_3_C coating underwent partial delamination with further formation of the grooves during the wear test at 23 °C. The delamination process was initiated at the cracks and the debris was crushed during the wear test as shown in Figure 10b. At 200 °C, the shape of debris indicated a lower degree of separation and delamination of the coating compared to that at 23 °C.

**Figure 11:**
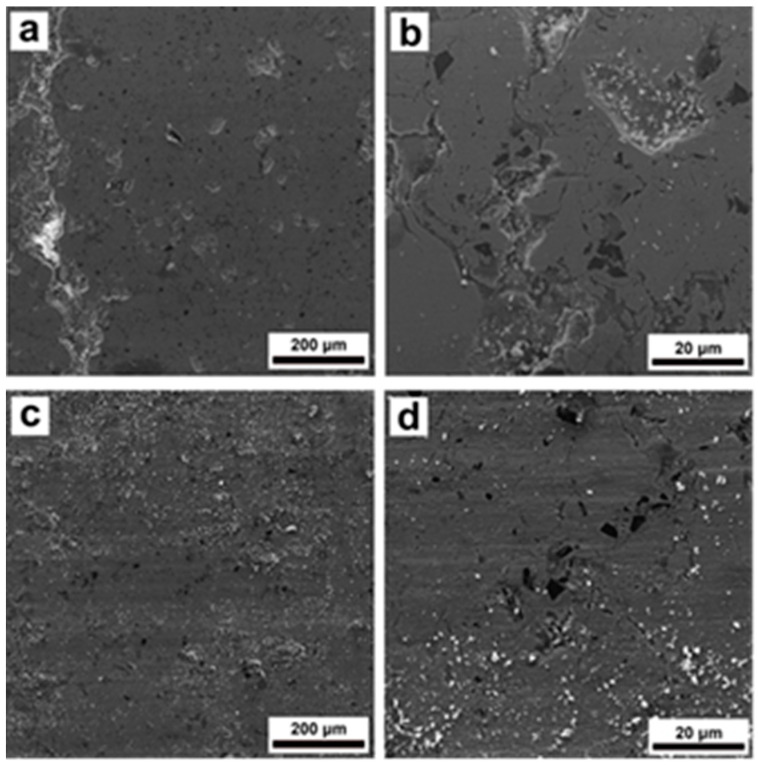
SEM micrographs of the wear tracks after wear tests of DGS deposited St-SiC at (**a**,**b**) 23 °C, and (**c**,**d**) 200 °C.

Figure 11a–d presents the wear tracks of the St-SiC coating at both temperatures. St-SiC coating experienced less deterioration compared to the other two types of coatings. However, some cracks on the wear surface at 23 °C were still formed, shown in Figure 11a. Higher magnification image in Figure 11b shows some microcracks on the surface of worn regions. A large number of debris particles remained on the wear tracks after the tests at 200 °C (Figure 11c,d), similar to the wear surface of the St-Fe_3_C coating in Figure 10c,d.

## 4. Discussion

The detailed comparison of the XRD patterns of the feedstock powders and coatings revealed that the DGS deposition process led to the formation of partially or almost fully amorphous structure in the coatings (Figure 1). It is worth mentioning that steel and St-SiC coatings demonstrated almost fully amorphous structure with small clearly distinguished peaks from crystalline phases.

The formation of partially and/or fully amorphous structure in the coatings is related to the high cooling rate of molten and/or semi-molten particles upon their impact with the substrate [22,35,38]. During the deposition process, accelerated molten particles with high in-flight velocity impact the substrate and result in the formation of thin splats. The process can be followed by large thermal gradient between the splats and substrate, which leads to rapid solidification of the sprayed particles [38]. Several works have reported that the in-flight velocities of particles during the DGS deposition process are in the range of 600–800 m/s [15,16,20]. The in-flight particle velocity can be highly affected by powder size. In the present work, the in-flight velocities of the particles were estimated to be 700–800 m/s for the average powder sizes in the range of 14–30 μm, where the lowest in-flight velocity corresponds to the larger particles [16]. 

It is well known that the rapid solidification suppresses the formation of crystalline phases and the formation of amorphous structure becomes preferential [34,39]. Similar behavior was observed for other iron-based alloys deposited using different thermal-spraying techniques [20,21,22,24,25,36,40,41]. While it was not in the scope of this study, it will be important in future studies to investigate the degree of amorphization in the coating samples produced by the DGS deposition technique. Generally, porosity has the strongest effect on the hardness of the coatings [12,30]. Thus, it is expected that an increase in the porosity level may result in an almost linear decrease in hardness regardless of the deposition process [12,42]. Nevertheless, the experimental results of this study do not indicate such a linear relationship between hardness and porosity (Figure 8). In particular, the hardest coating sample had the highest porosity level as well. Such findings indicated that porosity was not the only factor influencing the hardness of DGS-deposited coatings. The hardness is also affected by the coating structure, morphology, and phase distribution. To this end it is not a wrong assumption that adding hard particles could result in an increase in hardness of materials; however, opposite results were obtained in this study. More specifically, since ceramic particles remained solid during the DGS deposition due to their high melting temperature (approximately 2730 °C and 3140 °C for SiC and Fe_3_C) and relatively low spraying temperature (1200–1400 °C), the wettability of the solid particles and the bonding between particles and solidifying steel matrix were negatively affected. These small ceramic particles could fill the voids and therefore reduce the total porosity level of the coating; however, their unmelted state did not lead to strong bonding with the matrix and, consequently, caused lower hardness of the composites. The microstructures of both composite coatings (St-Fe_3_C and St-Sic) had lower porosity levels but lower hardness. Fe_3_C particles are larger with a higher melting temperature compared to the smaller SiC particles. There are more unmelted iron oxide particles within the microstructure of St-Fe_3_C, which lowered the bonding strength in this type of composite coating.

Wear resistances of all the coating samples were also examined at ambient (23 °C) and an elevated temperature of 200 °C. The unreinforced steel coating with the highest hardness (Figure 8 and Table 5) had the higher wear resistance, i.e., lower wear rate (0.13 mm^3^/h at 23 °C and 0.03 mm^3^/h at 200 °C). Such direct dependence of wear rate on hardness closely matched the typical behavior observed for other thermally sprayed coatings [20,43,44,45]. Thus, the hardness was considered as one of the main factors that affected the wear rate along with the hardness/elastic modulus ratio and residual stress [20].

The friction coefficient and wear rate of coatings have also been influenced by the wear test’s operating temperature. However, a nonlinear dependency between the temperature and friction coefficient was observed from the experiments. In particular, for the steel coating, an increase in the test temperature led to a significant decrease in the friction coefficient from 0.564 to 0.04 and wear rate from 0.13 mm^3^/h to 0.03 mm^3^/h. This behavior will be investigated in a future work, since no literature has previously reported such a decrease in wear rate in response to increasing test temperatures.

The mechanical properties, in particular, hardness, also significantly depend on the test temperature, i.e., an increase in temperature led to the reduction of hardness. As it was mentioned earlier, the hardness was established as the main contributor to the high wear resistance. Consequently, the degradation of the wear resistance was expected with the increase in temperature. This was indeed observed for reinforced composite coating samples in this study. The wear rate changed significantly from 0.15 mm^3^/h to 0.59 mm^3^/h and 0.17 mm^3^/h to 0.52 mm^3^/h for the St-Fe_3_C and St-SiC coatings, respectively. Similar behavior has previously been reported for Ni-based coating reinforced by SiC and iron-based coatings reinforced by WC [15,46]. Therefore, the addition of Fe_3_C and SiC particles led to the significant degradation of the wear resistance at a higher temperature compared to unreinforced steel coating. 

Visual observation of the wear tracks of the samples also helped to better understand the wear mechanism in each sample. It was clear from Figure 9, Figure 10 and Figure 11 that the presence of microcracks on the coating surfaces acted as precursors for initiating brittle cracking and further spallation and delamination of the coating fragments from the surface. Usually, the shear stress resulted from the normal load and friction force were responsible for such a phenomenon during the wear test [14,47]. Detailed observations of Figure 9, Figure 10 and Figure 11 indicate that after spallation and delamination in the coatings, further impact of the ceramic ball caused cracking and complete destruction of these fragments together with the formation of much debris. In turn, these damaged fragments promoted the abrasive wear of coatings during the wear test. It is worth mentioning that the steel coating possessed the lowest degree of fragmentation and damage compared to the other composite coatings, which could be associated with its higher hardness. Fracture or separation of ceramic particles from steel matrix due to low adhesion as previously discussed for hardness can also be a reason for the higher amount of debris in St-Fe_3_C and St-SiC coatings after wear test as shown in Figure 9b, Figure 10b and Figure 11b. An increase in the test temperature has also promoted the spallation and delamination on the coating surfaces of St-Fe_3_C and St-SiC samples.

## 5. Conclusions

The DGS method was proven to be a suitable technique for deposition of dense coatings with low porosity and relatively high thicknesses from metallic powders. The highest porosity of approximately 5 vol.% was observed for steel coatings and the porosities for St-Fe_3_C and St-SiC composites coatings were about 2.5 vol.% and 1 vol.%, respectively. 

It is a common assumption that ceramic reinforced composite materials should have higher hardness due to their lower porosity content. In this study, however, the steel coating had the highest hardness despite of having the highest porosity among all coatings, possibly due to the formation of amorphous structure as a result of rapid solidification of molten particles during the coating deposition. In cases of St-Fe_3_C and St-SiC coatings, the small ceramic particles could have filled the voids and showed lower porosity content in the image analysis; however, the lower bonding quality between the particles and steel matrix might have negatively affected the hardness. 

The hardness was a main factor that may affect the wear rate of thermally sprayed coatings, i.e., the harder steel coating possessed higher wear resistance. An increase in the test temperature resulted in a sharp decrease in friction coefficients of steel and St-SiC coatings. The same trend was also detected for the wear rate of the steel coating when the test temperature was increased to 200 °C. However, the addition of Fe_3_C and SiC in steel alloy led to a significant increase in the wear rate at the higher temperature condition. The softening of both coatings was responsible for the increased wear rate at high temperatures. Finally, it must be mentioned that, although it was speculated that the addition of harder materials to steel coating could increase the average hardness and lead to better wear resistance, the results obtained in this study showed the opposite outcome. The steel coating exhibited better mechanical properties (hardness and wear resistance) compared to St-Fe_3_C and St-SiC composite coatings. Future studies should further investigate how the quantity of ceramic particles will impact hardness of the composite manufactured by the DGS technology. 

## Figures and Tables

**Table 1:** Operational spraying parameters used for Detonation Gun Spraying (DGS).

Parameters	Value
Composition of explosive working gas mixture (acetylene/oxygen)	1.2/1.0
Filling degree of detonation gun barrel by working gas mixture	65%
Powder loading depth	400 mm
Frequency of shots	4 cycle/s
Barrel length	1000 mm
Spraying distance	120 mm
The rate of the substrate moving	5 mm/s

**Table 2:** Results of EDS analysis of steel powder and coating.

Points	Elements, wt.%						
	Fe	Cr	Mo	W	Si	C	Mn
**Powder (Figure 2)**							
A	55.67	18.02	13.71	6.41	3.85	0.50	1.83
B	58.56	19.91	14.93	-	3.60	-	2.55
C	53.53	18.16	16.24	5.35	3.94	0.80	1.98
D	53.75	18.51	15.80	5.43	3.70	0.57	2.25
E	54.12	18.71	14.82	5.51	3.73	0.51	1.89
**DGS Deposited Coating (Figure 3)**							
1	58.09	18.55	15.84	3.03	-	3.23	1.26
2	56.15	18.23	15.63	5.94	-	3.29	0.76
3	57.36	17.85	13.48	2.46	3.83	3.13	1.90
4	50.84	15.77	14.78	11.45	3.21	3.06	0.90
5	52.28	16.55	16.70	8.52	3.21	2.75	-
6	53.72	18.03	15.04	5.02	3.94	2.86	1.38
7	32.18	21.47	28.45	12.58	-	3.87	0.86

**Table 3:** Results of EDS analysis on St-Fe_3_C powder and coating.

Points	Elements, wt.%						
	Fe	Cr	Mo	W	Si	C	Mn
**Powder**							
F	56.16	18.16	15.60	4.67	4.16	1.25	-
G	54.84	19.40	14.65	3.91	3.99	1.33	1.87
H	95.23	-	-	6.74	0.59	2.19	1.01
I	97.49	-	-	-	0.82	0.76	0.93
J	51.47	17.52	16.06	7.91	3.98	1.25	1.81
**Coating**							
1	96.58	1.08	-	-	-	2.34	-
2	58.19	18.54	15.60	-	3.93	2.12	1.62
3	49.81	18.57	17.94	7.59	3.88	2.21	-
4	90.49	3.64	2.91	-	1.37	1.59	-
5	90.36	3.0	2.98	-	1.42	1.77	-
6	77.64	7.28	7.26	4.75	-	2.07	1.01
7	-	-	-	95.53	-	4.47	-

**Table 4:** Results of EDS analysis on St-SiC powder and coating.

Points	Elements, wt.%						
	Fe	Cr	Mo	W	Si	C	Mn
**Powder**							
K	54.41	18.48	17.28	-	5.52	1.71	1.51
L	-	-	-	-	95.93	4.07	-
M	-	-	-	-	93.25	6.75	-
N	55.45	18.71	14.81	3.47	4.23	1.41	1.92
O	52.16	17.73	14.73	7.44	4.95	1.06	1.94
**Coating**							
1	-	-	-	-	88.14	11.86	-
2	51.38	18.28	15.25	5.69	4.04	3.51	1.85
3	-	-	-	-	87.83	12.06	-
4	50.73	18.01	14.97	5.11	4.62	3.87	1.31
5	48.95	17.48	16.79	5.83	4.14	4.12	2.06
6	-	-	-	-	88.33	11.91	-
7	51.41	18.11	14.99	5.47	4.22	3.93	1.73

**Table 5:** The comparison between predicted hardness and actual measured harnesses in this study.

Coating	Predicted Hardness *	Measured Hardness
	(HV)	(HV)
Steel	~1000 HV	1021 ± 60.80
St-Fe_3_C	~1200 HV	830 ± 61
St-SiC	~1700 HV	847 ± 96

* The predicted values are approximated due to different range of reported hardness for SHS 7475 alloy.

**Table 6:** Friction coefficients and wear rates (at 23 °C and at 200 °C) of (1) Steel, (2) St-Fe_3_C, and (3) St-SiC coatings deposited by DGS.

Coating	Friction Coefficient		Wear Rate (mm^3^/h)	
	23°	200°	23°	200°
Steel	0.564	0.04	0.13	0.03
St-Fe_3_C	0.479	0.584	0.15	0.59
St-SiC	0.745	0.657	0.17	0.52

## Data Availability

Extra research data is available upon direct request from the authors.
